# Crystal structure of bis­{2,4-di-*tert*-butyl-6-[(iso­propyl­imino)­meth­yl]phenolato-κ^2^ *N*,*O*}zinc di­chloro­methane mono­solvate

**DOI:** 10.1107/S1600536814022636

**Published:** 2014-11-05

**Authors:** Yuan-Zeng Hao

**Affiliations:** a Guangzhou Super-Dragon Engineering Plastics Co. Ltd, People’s Republic of China; b Guangzhou Engineering, Technology Research Center, Guangzhou 510900, People’s Republic of China

**Keywords:** crystal structure, Schiff base, zinc, tetra­hedral coordination

## Abstract

In the title compound, [Zn(C_18_H_28_NO)_2_]·CH_2_Cl_2_, the Zn^II^ atom is *N*,*O*-chelated by two crystallographically independent salicyl­aldehyde imine ligands, leading to a distorted tetra­hedral coordination sphere. The dihedral angle between the planes of the two metallacycles is 88.69 (6)°. Intra­molecular non-classical C—H⋯O hydrogen-bonding inter­actions are observed. In the crystal, the complex mol­ecules stack into columns along the *a* axis. Di­chloro­methane solvent mol­ecules are situated in the voids of this arrangement.

## Related literature   

For backgroud to poly(lactide) (PLA) and its copolymers, see: Wheaton & Hayes (2011 ▶); Chen *et al.* (2006 ▶). For the use of bulky ligands coordinating to the active metal site to avoid undesirable transesterification during synthesis of lactides by ring-opening polymerization (ROP), see: Wu *et al.* (2006 ▶). For a highly active zinc catalyst for the controlled polymerization of lactides, see: Williams *et al.* (2003 ▶); Chamberlain *et al.* (2001 ▶). For the preparation of zinc salicyl­aldehyde­imine complexes, see: Chisholm *et al.* (2001 ▶).
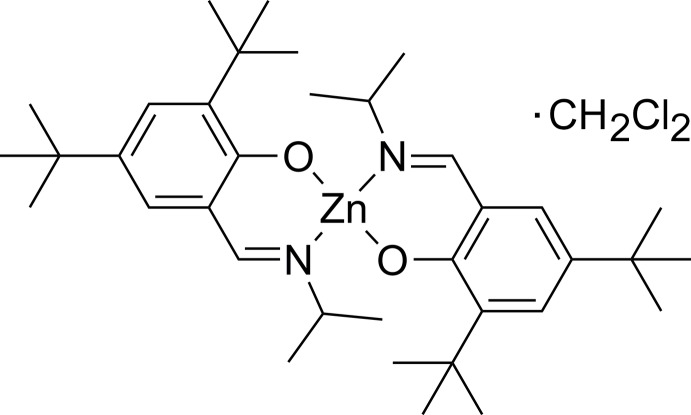


## Experimental   

### Crystal data  

[Zn(C_18_H_28_NO)_2_]·CH_2_Cl_2_
*M* *_r_* = 699.12
Monoclinic, 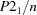

*a* = 13.6653 (17) Å
*b* = 14.6674 (18) Å
*c* = 19.663 (2) Å
β = 104.807 (2)°
*V* = 3810.4 (8) Å^3^
*Z* = 4
Mo *K*α radiation
μ = 0.82 mm^−1^
*T* = 173 K
0.42 × 0.41 × 0.26 mm

### Data collection  

Bruker APEXII area-detector diffractometer
Absorption correction: multi-scan (*SADABS*; Bruker, 2007 ▶) *T*_min_ = 0.725, *T*_max_ = 0.816
22339 measured reflections
8281 independent reflections
6513 reflections with *I* > 2σ(*I*)
*R*_int_ = 0.024

### Refinement  

*R*[*F* ^2^ > 2σ(*F* ^2^)] = 0.036
*wR*(*F* ^2^) = 0.101
*S* = 1.03
8281 reflections
413 parameters
H-atom parameters constrained
Δρ_max_ = 0.52 e Å^−3^
Δρ_min_ = −0.37 e Å^−3^

### 

Data collection: *APEX2* (Bruker, 2007 ▶); cell refinement: *SAINT* (Bruker, 2007 ▶); data reduction: *SAINT*; program(s) used to solve structure: *SHELXS97* (Sheldrick, 2008 ▶); program(s) used to refine structure: *SHELXL97* (Sheldrick, 2008 ▶); molecular graphics: *SHELXTL* (Sheldrick, 2008 ▶); software used to prepare material for publication: *publCIF* (Westrip, 2010 ▶).

## Supplementary Material

Crystal structure: contains datablock(s) I, New_Global_Publ_Block. DOI: 10.1107/S1600536814022636/wm5038sup1.cif

Structure factors: contains datablock(s) I. DOI: 10.1107/S1600536814022636/wm5038Isup2.hkl

**Fig. 1.**
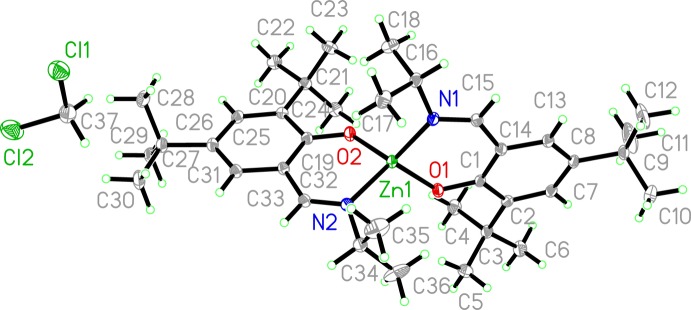
The molecular structure of the title complex with atom labelling and displacement ellipsoids drawn at the 30% probability level.

. DOI: 10.1107/S1600536814022636/wm5038fig1.tif
The mol­ecular structure of the title complex with atom labelling and displacement ellipsoids drawn at the 30% probability level.

**Fig. 2.**
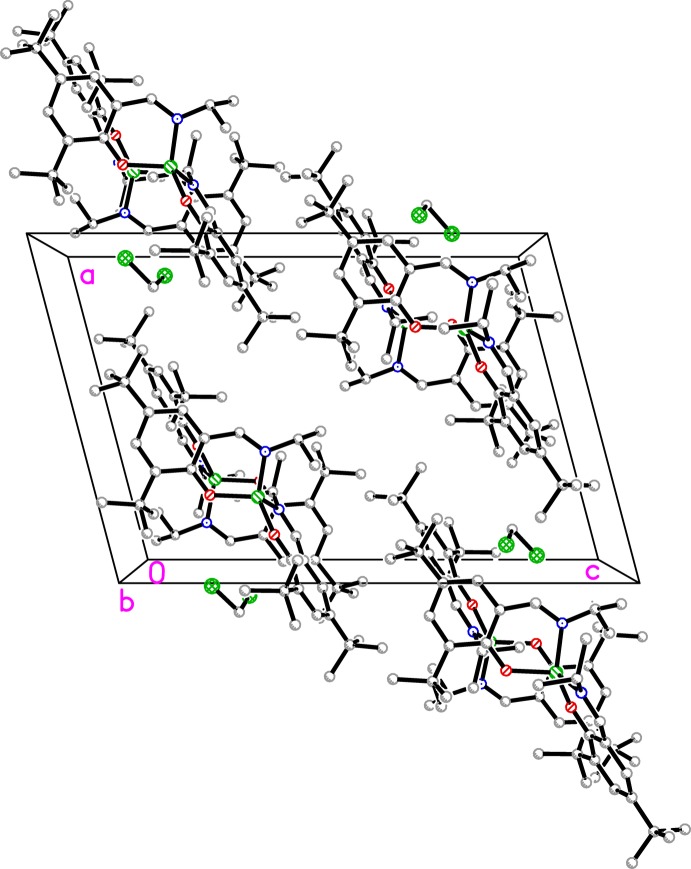
View of the crystal structure of title compound; H atoms are omitted for clarity.

. DOI: 10.1107/S1600536814022636/wm5038fig2.tif
View of the crystal structure of title compound; H atoms are omitted for clarity.

CCDC reference: 1029220

Additional supporting information:  crystallographic information; 3D view; checkCIF report

## Figures and Tables

**Table 1:** Hydrogen-bond geometry (, )

*D*H*A*	*D*H	H*A*	*D* *A*	*D*H*A*
C4H4*A*O1	0.98	2.37	3.018(3)	123
C5H5*C*O1	0.98	2.32	2.967(3)	123
C23H23*C*O2	0.98	2.35	2.994(3)	122
C24H24*A*O2	0.98	2.33	2.986(3)	124

## supplementary crystallographic information

### S1. Experimental

Synthesis of the ligand: Diisopropylamine (2.02 g, 20 mmol) was added dropwise to a solution of the 2,6-di-*tert*-butyl-salicylaldehyde (4.68 g, 20 mmol) in dry ethanol (60 ml) at room temperature over a period of 5 min. The mixture was stirred at 353 K for four hours. Then the solvent was removed by rotary evaporation, and the residue was recrystallizated from methanol. The ligand was isolated as a yellow solid in 20% yield.

Synthesis of the complex: In a Schlenk flask, ZnEt_2_ (1.22 g, 10 mmol) was added to the solution of the salicylaldehydeimine ligand (10 mmol in tetrahydrofuran) at room temperature. The reaction mixture was stirred in the absence of light for 3 hours at room temperature and was then filtered in the dark and the volume of the solution reduced to 5.0 ml. Pentane was added to afford the product as a light-green solid in *ca*. 50% yield. Single-crystals suitable for X-ray diffraction were grown by slow evaporation of a solution of the title compound in dichlormethane at room temperature.

### S2. Refinement

Reflection (011) was affected by the beamstop and was omitted from the refinement. H atoms were placed in geometrically idealized positions and constrained to ride on their parent atoms with a C—H distances of 0.95 (aromatic) and 0.99 Å (methylene) and with *U*_iso_(H) = 1.2*U*_eq_(C), and 0.98 Å for CH_3_ [*U*_iso_(H)= 1.5*U*_eq_(C)].

### Crystal data

**Table:**

[Zn(C_18_H_28_NO)_2_]·CH_2_Cl_2_	*F*(000) = 1496
*M**_r_* = 699.12	*D*_x_ = 1.219 Mg m^−^^3^
Monoclinic, *P*2_1_/*n*	Mo *K*α radiation, λ = 0.71073 Å
Hall symbol: -P 2yn	Cell parameters from 5049 reflections
*a* = 13.6653 (17) Å	θ = 2.1–27.0°
*b* = 14.6674 (18) Å	µ = 0.82 mm^−^^1^
*c* = 19.663 (2) Å	*T* = 173 K
β = 104.807 (2)°	Block, light-green
*V* = 3810.4 (8) Å^3^	0.42 × 0.41 × 0.26 mm
*Z* = 4	

### Data collection

**Table:**

Bruker APEXII area-detector diffractometer	8281 independent reflections
Radiation source: fine-focus sealed tube	6513 reflections with *I* > 2σ(*I*)
Graphite monochromator	*R*_int_ = 0.024
φ and ω scans	θ_max_ = 27.1°, θ_min_ = 1.8°
Absorption correction: multi-scan (*SADABS*; Bruker, 2007)	*h* = −17→8
*T*_min_ = 0.725, *T*_max_ = 0.816	*k* = −18→18
22339 measured reflections	*l* = −25→25

### Refinement

**Table:**

Refinement on *F*^2^	Primary atom site location: structure-invariant direct methods
Least-squares matrix: full	Secondary atom site location: difference Fourier map
*R*[*F*^2^ > 2σ(*F*^2^)] = 0.036	Hydrogen site location: inferred from neighbouring sites
*wR*(*F*^2^) = 0.101	H-atom parameters constrained
*S* = 1.03	*w* = 1/[σ^2^(*F*_o_^2^) + (0.0501*P*)^2^ + 2.0294*P*] where *P* = (*F*_o_^2^ + 2*F*_c_^2^)/3
8281 reflections	(Δ/σ)_max_ < 0.001
413 parameters	Δρ_max_ = 0.52 e Å^−^^3^
0 restraints	Δρ_min_ = −0.37 e Å^−^^3^

### Special details

**Table:**

Geometry. All esds (except the esd in the dihedral angle between two l.s. planes) are estimated using the full covariance matrix. The cell esds are taken into account individually in the estimation of esds in distances, angles and torsion angles; correlations between esds in cell parameters are only used when they are defined by crystal symmetry. An approximate (isotropic) treatment of cell esds is used for estimating esds involving l.s. planes.
Refinement. Refinement of *F*^2^ against ALL reflections. The weighted *R*-factor *wR* and goodness of fit *S* are based on *F*^2^, conventional *R*-factors *R* are based on *F*, with *F* set to zero for negative *F*^2^. The threshold expression of *F*^2^ > σ(*F*^2^) is used only for calculating *R*-factors(gt) *etc*. and is not relevant to the choice of reflections for refinement. *R*-factors based on *F*^2^ are statistically about twice as large as those based on *F*, and *R*- factors based on ALL data will be even larger.

### Fractional atomic coordinates and isotropic or equivalent isotropic displacement parameters (Å^2^)

**Table:**

	*x*	*y*	*z*	*U*_iso_*/*U*_eq_	
Zn1	0.231421 (16)	0.615892 (14)	0.299369 (11)	0.02227 (7)	
O1	0.12145 (10)	0.68715 (9)	0.31431 (7)	0.0271 (3)	
N2	0.36935 (12)	0.66973 (11)	0.33979 (8)	0.0236 (3)	
N1	0.18811 (12)	0.49721 (10)	0.33244 (8)	0.0232 (3)	
O2	0.23720 (10)	0.61964 (10)	0.20306 (7)	0.0276 (3)	
C1	0.04377 (14)	0.65800 (13)	0.33501 (9)	0.0220 (4)	
C19	0.31561 (14)	0.63896 (12)	0.17944 (10)	0.0222 (4)	
C2	−0.03878 (15)	0.71885 (13)	0.33494 (9)	0.0236 (4)	
C7	−0.11968 (15)	0.68601 (13)	0.35778 (10)	0.0251 (4)	
H7	−0.1738	0.7269	0.3573	0.030*	
C14	0.03560 (14)	0.56642 (13)	0.35720 (9)	0.0228 (4)	
C13	−0.04928 (15)	0.53872 (13)	0.38081 (10)	0.0244 (4)	
H13	−0.0519	0.4778	0.3967	0.029*	
C15	0.10583 (14)	0.49406 (13)	0.35310 (9)	0.0238 (4)	
H15	0.0889	0.4357	0.3678	0.029*	
C21	0.20705 (15)	0.60533 (13)	0.05374 (10)	0.0264 (4)	
C16	0.23887 (15)	0.40931 (13)	0.32679 (11)	0.0276 (4)	
H16	0.2070	0.3606	0.3496	0.033*	
C18	0.22391 (19)	0.38570 (15)	0.24970 (12)	0.0389 (5)	
H18A	0.1513	0.3822	0.2268	0.058*	
H18B	0.2556	0.3267	0.2456	0.058*	
H18C	0.2551	0.4329	0.2269	0.058*	
C24	0.12496 (16)	0.67508 (15)	0.05796 (11)	0.0333 (5)	
H24A	0.1176	0.6775	0.1062	0.050*	
H24B	0.0604	0.6569	0.0260	0.050*	
H24C	0.1444	0.7354	0.0444	0.050*	
C33	0.42696 (14)	0.68492 (13)	0.29838 (10)	0.0242 (4)	
H33	0.4907	0.7113	0.3202	0.029*	
C34	0.40723 (16)	0.69940 (14)	0.41395 (10)	0.0295 (4)	
H34	0.4714	0.7344	0.4183	0.035*	
C23	0.17481 (18)	0.50939 (14)	0.07091 (11)	0.0355 (5)	
H23A	0.2276	0.4654	0.0681	0.053*	
H23B	0.1114	0.4926	0.0370	0.053*	
H23C	0.1648	0.5089	0.1185	0.053*	
C3	−0.03675 (15)	0.81732 (13)	0.30842 (11)	0.0278 (4)	
Cl1	0.91415 (5)	0.39983 (5)	0.22012 (4)	0.05363 (17)	
Cl2	0.95759 (7)	0.56715 (6)	0.15546 (4)	0.0742 (3)	
C6	−0.13092 (18)	0.87132 (14)	0.31363 (14)	0.0397 (5)	
H6A	−0.1917	0.8419	0.2846	0.060*	
H6B	−0.1264	0.9337	0.2969	0.060*	
H6C	−0.1347	0.8728	0.3627	0.060*	
C20	0.30799 (14)	0.63163 (12)	0.10503 (9)	0.0216 (4)	
C31	0.49349 (15)	0.68533 (13)	0.19565 (10)	0.0255 (4)	
H31	0.5554	0.7049	0.2264	0.031*	
C22	0.21423 (18)	0.60298 (16)	−0.02269 (11)	0.0361 (5)	
H22A	0.2368	0.6625	−0.0354	0.054*	
H22B	0.1476	0.5888	−0.0537	0.054*	
H22C	0.2629	0.5561	−0.0279	0.054*	
C25	0.39349 (15)	0.64874 (13)	0.08192 (10)	0.0255 (4)	
H25	0.3882	0.6425	0.0330	0.031*	
C8	−0.12791 (15)	0.59650 (13)	0.38169 (10)	0.0244 (4)	
C9	−0.22116 (15)	0.56725 (14)	0.40596 (11)	0.0284 (4)	
C26	0.48822 (15)	0.67477 (13)	0.12575 (10)	0.0258 (4)	
C4	−0.03558 (18)	0.81635 (15)	0.23040 (11)	0.0360 (5)	
H4A	0.0233	0.7818	0.2249	0.054*	
H4B	−0.0316	0.8790	0.2141	0.054*	
H4C	−0.0977	0.7876	0.2025	0.054*	
C35	0.4302 (3)	0.61854 (19)	0.46227 (13)	0.0566 (8)	
H35A	0.3677	0.5845	0.4599	0.085*	
H35B	0.4589	0.6395	0.5106	0.085*	
H35C	0.4790	0.5789	0.4479	0.085*	
C17	0.35026 (17)	0.41501 (16)	0.36411 (12)	0.0381 (5)	
H17A	0.3823	0.4626	0.3422	0.057*	
H17B	0.3827	0.3562	0.3605	0.057*	
H17C	0.3579	0.4299	0.4138	0.057*	
C5	0.05595 (18)	0.86917 (14)	0.35161 (12)	0.0374 (5)	
H5A	0.0538	0.8711	0.4010	0.056*	
H5B	0.0555	0.9315	0.3335	0.056*	
H5C	0.1178	0.8380	0.3479	0.056*	
C30	0.67305 (17)	0.72050 (19)	0.14855 (12)	0.0439 (6)	
H30A	0.6591	0.7790	0.1683	0.066*	
H30B	0.7289	0.7279	0.1262	0.066*	
H30C	0.6917	0.6752	0.1863	0.066*	
C27	0.57872 (15)	0.68851 (14)	0.09393 (11)	0.0295 (4)	
C32	0.40933 (14)	0.66789 (13)	0.22385 (9)	0.0230 (4)	
C37	0.87357 (19)	0.51175 (18)	0.19483 (13)	0.0437 (6)	
H37A	0.8058	0.5096	0.1614	0.052*	
H37B	0.8678	0.5466	0.2368	0.052*	
C29	0.55214 (17)	0.76021 (15)	0.03504 (12)	0.0358 (5)	
H29A	0.5363	0.8183	0.0545	0.054*	
H29B	0.4933	0.7396	−0.0015	0.054*	
H29C	0.6099	0.7685	0.0146	0.054*	
C28	0.60274 (19)	0.59837 (15)	0.06249 (13)	0.0398 (5)	
H28A	0.6606	0.6068	0.0422	0.060*	
H28B	0.5437	0.5786	0.0256	0.060*	
H28C	0.6193	0.5519	0.0995	0.060*	
C36	0.3316 (2)	0.7614 (2)	0.43351 (13)	0.0629 (9)	
H36A	0.3173	0.8129	0.4007	0.094*	
H36B	0.3590	0.7841	0.4815	0.094*	
H36C	0.2688	0.7277	0.4311	0.094*	
C10	−0.2250 (3)	0.6212 (2)	0.47101 (18)	0.0754 (11)	
H10A	−0.2275	0.6865	0.4601	0.113*	
H10B	−0.1645	0.6081	0.5089	0.113*	
H10C	−0.2856	0.6040	0.4860	0.113*	
C12	−0.2184 (2)	0.46734 (19)	0.4255 (2)	0.0779 (11)	
H12A	−0.2801	0.4516	0.4397	0.117*	
H12B	−0.1591	0.4555	0.4646	0.117*	
H12C	−0.2143	0.4303	0.3849	0.117*	
C11	−0.3154 (2)	0.5852 (3)	0.34809 (19)	0.0842 (12)	
H11A	−0.3750	0.5660	0.3635	0.126*	
H11B	−0.3123	0.5506	0.3060	0.126*	
H11C	−0.3203	0.6504	0.3371	0.126*	

### Atomic displacement parameters (Å^2^)

**Table:**

	*U*^11^	*U*^22^	*U*^33^	*U*^12^	*U*^13^	*U*^23^
Zn1	0.02082 (12)	0.02398 (12)	0.02386 (12)	−0.00122 (9)	0.00912 (9)	0.00045 (8)
O1	0.0244 (7)	0.0247 (7)	0.0362 (7)	−0.0010 (5)	0.0151 (6)	0.0008 (6)
N2	0.0242 (8)	0.0261 (8)	0.0197 (7)	−0.0019 (7)	0.0038 (6)	0.0007 (6)
N1	0.0239 (8)	0.0229 (8)	0.0248 (8)	0.0020 (6)	0.0098 (7)	0.0011 (6)
O2	0.0219 (7)	0.0388 (8)	0.0227 (7)	−0.0043 (6)	0.0069 (5)	−0.0003 (6)
C1	0.0219 (9)	0.0256 (9)	0.0192 (8)	−0.0018 (7)	0.0067 (7)	−0.0016 (7)
C19	0.0215 (9)	0.0208 (9)	0.0246 (9)	0.0003 (7)	0.0061 (7)	0.0019 (7)
C2	0.0261 (10)	0.0232 (9)	0.0221 (9)	0.0012 (8)	0.0075 (8)	−0.0015 (7)
C7	0.0241 (10)	0.0260 (9)	0.0267 (9)	0.0033 (8)	0.0092 (8)	−0.0021 (8)
C14	0.0214 (9)	0.0249 (9)	0.0226 (9)	0.0001 (7)	0.0064 (7)	−0.0004 (7)
C13	0.0268 (10)	0.0243 (9)	0.0234 (9)	−0.0007 (8)	0.0089 (8)	0.0018 (7)
C15	0.0264 (10)	0.0223 (9)	0.0233 (9)	−0.0011 (7)	0.0076 (8)	0.0028 (7)
C21	0.0268 (10)	0.0279 (10)	0.0230 (9)	−0.0014 (8)	0.0035 (8)	−0.0007 (8)
C16	0.0266 (10)	0.0233 (9)	0.0365 (11)	0.0045 (8)	0.0149 (9)	0.0039 (8)
C18	0.0449 (13)	0.0316 (11)	0.0401 (12)	0.0075 (10)	0.0108 (10)	−0.0073 (9)
C24	0.0287 (11)	0.0368 (11)	0.0313 (10)	0.0033 (9)	0.0020 (9)	0.0015 (9)
C33	0.0216 (9)	0.0266 (9)	0.0236 (9)	−0.0022 (8)	0.0043 (8)	0.0012 (7)
C34	0.0287 (11)	0.0395 (11)	0.0191 (9)	−0.0070 (9)	0.0039 (8)	−0.0025 (8)
C23	0.0374 (12)	0.0312 (11)	0.0344 (11)	−0.0091 (9)	0.0026 (9)	−0.0014 (9)
C3	0.0294 (11)	0.0239 (9)	0.0332 (10)	0.0031 (8)	0.0133 (9)	0.0020 (8)
Cl1	0.0483 (4)	0.0510 (4)	0.0571 (4)	−0.0032 (3)	0.0053 (3)	−0.0029 (3)
Cl2	0.0979 (6)	0.0661 (5)	0.0702 (5)	−0.0349 (4)	0.0430 (5)	−0.0148 (4)
C6	0.0418 (13)	0.0267 (11)	0.0575 (15)	0.0087 (9)	0.0253 (12)	0.0071 (10)
C20	0.0221 (9)	0.0211 (9)	0.0208 (8)	0.0005 (7)	0.0042 (7)	0.0008 (7)
C31	0.0221 (9)	0.0295 (10)	0.0243 (9)	−0.0024 (8)	0.0048 (8)	0.0009 (8)
C22	0.0363 (12)	0.0459 (13)	0.0236 (10)	−0.0045 (10)	0.0033 (9)	−0.0043 (9)
C25	0.0288 (10)	0.0283 (9)	0.0204 (9)	0.0001 (8)	0.0080 (8)	0.0006 (8)
C8	0.0233 (10)	0.0293 (10)	0.0227 (9)	−0.0002 (8)	0.0097 (8)	−0.0013 (7)
C9	0.0246 (10)	0.0299 (10)	0.0353 (11)	0.0005 (8)	0.0159 (9)	0.0018 (8)
C26	0.0250 (10)	0.0279 (10)	0.0267 (9)	−0.0002 (8)	0.0105 (8)	0.0023 (8)
C4	0.0406 (13)	0.0345 (11)	0.0354 (11)	0.0045 (10)	0.0142 (10)	0.0080 (9)
C35	0.079 (2)	0.0570 (16)	0.0265 (12)	0.0163 (15)	0.0008 (13)	0.0044 (11)
C17	0.0335 (12)	0.0380 (12)	0.0433 (13)	0.0112 (10)	0.0108 (10)	0.0025 (10)
C5	0.0428 (13)	0.0269 (11)	0.0440 (13)	−0.0043 (9)	0.0140 (11)	−0.0028 (9)
C30	0.0271 (12)	0.0695 (17)	0.0386 (12)	−0.0089 (11)	0.0146 (10)	−0.0034 (12)
C27	0.0255 (10)	0.0361 (11)	0.0295 (10)	−0.0017 (8)	0.0116 (8)	0.0005 (8)
C32	0.0224 (9)	0.0240 (9)	0.0229 (9)	−0.0010 (7)	0.0061 (7)	0.0017 (7)
C37	0.0384 (13)	0.0545 (15)	0.0381 (12)	0.0002 (11)	0.0093 (10)	−0.0028 (11)
C29	0.0393 (12)	0.0345 (11)	0.0385 (12)	−0.0044 (9)	0.0188 (10)	0.0030 (9)
C28	0.0398 (13)	0.0386 (12)	0.0483 (13)	0.0059 (10)	0.0244 (11)	0.0020 (10)
C36	0.076 (2)	0.0688 (19)	0.0339 (13)	0.0311 (16)	−0.0052 (13)	−0.0218 (13)
C10	0.091 (2)	0.082 (2)	0.078 (2)	−0.0284 (18)	0.067 (2)	−0.0262 (17)
C12	0.0624 (19)	0.0434 (15)	0.155 (3)	0.0052 (14)	0.077 (2)	0.0251 (18)
C11	0.0269 (14)	0.144 (3)	0.078 (2)	−0.0172 (17)	0.0057 (14)	0.044 (2)

### Geometric parameters (Å, º)

**Table:**

Zn1—O1	1.9138 (13)	C20—C25	1.380 (3)
Zn1—O2	1.9163 (13)	C31—C26	1.367 (3)
Zn1—N1	2.0001 (15)	C31—C32	1.422 (3)
Zn1—N2	2.0105 (16)	C31—H31	0.9500
O1—C1	1.302 (2)	C22—H22A	0.9800
N2—C33	1.289 (2)	C22—H22B	0.9800
N2—C34	1.483 (2)	C22—H22C	0.9800
N1—C15	1.290 (2)	C25—C26	1.412 (3)
N1—C16	1.482 (2)	C25—H25	0.9500
O2—C19	1.303 (2)	C8—C9	1.532 (3)
C1—C14	1.425 (3)	C9—C11	1.508 (4)
C1—C2	1.438 (3)	C9—C12	1.513 (3)
C19—C32	1.418 (3)	C9—C10	1.517 (3)
C19—C20	1.444 (3)	C26—C27	1.535 (3)
C2—C7	1.382 (3)	C4—H4A	0.9800
C2—C3	1.538 (3)	C4—H4B	0.9800
C7—C8	1.409 (3)	C4—H4C	0.9800
C7—H7	0.9500	C35—H35A	0.9800
C14—C13	1.415 (3)	C35—H35B	0.9800
C14—C15	1.447 (3)	C35—H35C	0.9800
C13—C8	1.372 (3)	C17—H17A	0.9800
C13—H13	0.9500	C17—H17B	0.9800
C15—H15	0.9500	C17—H17C	0.9800
C21—C22	1.531 (3)	C5—H5A	0.9800
C21—C20	1.535 (3)	C5—H5B	0.9800
C21—C24	1.536 (3)	C5—H5C	0.9800
C21—C23	1.538 (3)	C30—C27	1.526 (3)
C16—C17	1.514 (3)	C30—H30A	0.9800
C16—C18	1.517 (3)	C30—H30B	0.9800
C16—H16	1.0000	C30—H30C	0.9800
C18—H18A	0.9800	C27—C28	1.530 (3)
C18—H18B	0.9800	C27—C29	1.538 (3)
C18—H18C	0.9800	C37—H37A	0.9900
C24—H24A	0.9800	C37—H37B	0.9900
C24—H24B	0.9800	C29—H29A	0.9800
C24—H24C	0.9800	C29—H29B	0.9800
C33—C32	1.445 (3)	C29—H29C	0.9800
C33—H33	0.9500	C28—H28A	0.9800
C34—C36	1.499 (3)	C28—H28B	0.9800
C34—C35	1.502 (3)	C28—H28C	0.9800
C34—H34	1.0000	C36—H36A	0.9800
C23—H23A	0.9800	C36—H36B	0.9800
C23—H23B	0.9800	C36—H36C	0.9800
C23—H23C	0.9800	C10—H10A	0.9800
C3—C5	1.534 (3)	C10—H10B	0.9800
C3—C6	1.537 (3)	C10—H10C	0.9800
C3—C4	1.538 (3)	C12—H12A	0.9800
Cl1—C37	1.763 (3)	C12—H12B	0.9800
Cl2—C37	1.741 (3)	C12—H12C	0.9800
C6—H6A	0.9800	C11—H11A	0.9800
C6—H6B	0.9800	C11—H11B	0.9800
C6—H6C	0.9800	C11—H11C	0.9800
			
O1—Zn1—O2	111.62 (6)	H22B—C22—H22C	109.5
O1—Zn1—N1	96.67 (6)	C20—C25—C26	124.69 (17)
O2—Zn1—N1	115.89 (6)	C20—C25—H25	117.7
O1—Zn1—N2	114.73 (6)	C26—C25—H25	117.7
O2—Zn1—N2	96.21 (6)	C13—C8—C7	116.22 (17)
N1—Zn1—N2	122.59 (6)	C13—C8—C9	123.19 (17)
C1—O1—Zn1	127.12 (12)	C7—C8—C9	120.58 (17)
C33—N2—C34	117.05 (16)	C11—C9—C12	108.7 (3)
C33—N2—Zn1	118.72 (13)	C11—C9—C10	109.8 (3)
C34—N2—Zn1	124.10 (12)	C12—C9—C10	107.2 (2)
C15—N1—C16	117.05 (16)	C11—C9—C8	109.53 (18)
C15—N1—Zn1	119.19 (13)	C12—C9—C8	112.46 (17)
C16—N1—Zn1	123.36 (12)	C10—C9—C8	109.12 (19)
C19—O2—Zn1	127.14 (12)	C31—C26—C25	116.67 (17)
O1—C1—C14	122.61 (17)	C31—C26—C27	123.65 (18)
O1—C1—C2	119.85 (17)	C25—C26—C27	119.68 (17)
C14—C1—C2	117.53 (16)	C3—C4—H4A	109.5
O2—C19—C32	122.75 (17)	C3—C4—H4B	109.5
O2—C19—C20	119.47 (17)	H4A—C4—H4B	109.5
C32—C19—C20	117.78 (16)	C3—C4—H4C	109.5
C7—C2—C1	118.41 (17)	H4A—C4—H4C	109.5
C7—C2—C3	121.52 (17)	H4B—C4—H4C	109.5
C1—C2—C3	120.05 (16)	C34—C35—H35A	109.5
C2—C7—C8	124.90 (18)	C34—C35—H35B	109.5
C2—C7—H7	117.6	H35A—C35—H35B	109.5
C8—C7—H7	117.6	C34—C35—H35C	109.5
C13—C14—C1	120.40 (17)	H35A—C35—H35C	109.5
C13—C14—C15	114.75 (16)	H35B—C35—H35C	109.5
C1—C14—C15	124.70 (16)	C16—C17—H17A	109.5
C8—C13—C14	122.49 (17)	C16—C17—H17B	109.5
C8—C13—H13	118.8	H17A—C17—H17B	109.5
C14—C13—H13	118.8	C16—C17—H17C	109.5
N1—C15—C14	129.42 (17)	H17A—C17—H17C	109.5
N1—C15—H15	115.3	H17B—C17—H17C	109.5
C14—C15—H15	115.3	C3—C5—H5A	109.5
C22—C21—C20	112.22 (17)	C3—C5—H5B	109.5
C22—C21—C24	107.39 (17)	H5A—C5—H5B	109.5
C20—C21—C24	110.06 (16)	C3—C5—H5C	109.5
C22—C21—C23	106.78 (17)	H5A—C5—H5C	109.5
C20—C21—C23	110.21 (16)	H5B—C5—H5C	109.5
C24—C21—C23	110.09 (18)	C27—C30—H30A	109.5
N1—C16—C17	110.34 (17)	C27—C30—H30B	109.5
N1—C16—C18	109.12 (16)	H30A—C30—H30B	109.5
C17—C16—C18	111.04 (18)	C27—C30—H30C	109.5
N1—C16—H16	108.8	H30A—C30—H30C	109.5
C17—C16—H16	108.8	H30B—C30—H30C	109.5
C18—C16—H16	108.8	C30—C27—C28	108.68 (19)
C16—C18—H18A	109.5	C30—C27—C26	112.16 (17)
C16—C18—H18B	109.5	C28—C27—C26	109.24 (17)
H18A—C18—H18B	109.5	C30—C27—C29	108.32 (18)
C16—C18—H18C	109.5	C28—C27—C29	108.69 (17)
H18A—C18—H18C	109.5	C26—C27—C29	109.68 (17)
H18B—C18—H18C	109.5	C19—C32—C31	120.46 (16)
C21—C24—H24A	109.5	C19—C32—C33	124.51 (17)
C21—C24—H24B	109.5	C31—C32—C33	115.00 (17)
H24A—C24—H24B	109.5	Cl2—C37—Cl1	111.36 (14)
C21—C24—H24C	109.5	Cl2—C37—H37A	109.4
H24A—C24—H24C	109.5	Cl1—C37—H37A	109.4
H24B—C24—H24C	109.5	Cl2—C37—H37B	109.4
N2—C33—C32	129.84 (18)	Cl1—C37—H37B	109.4
N2—C33—H33	115.1	H37A—C37—H37B	108.0
C32—C33—H33	115.1	C27—C29—H29A	109.5
N2—C34—C36	109.88 (17)	C27—C29—H29B	109.5
N2—C34—C35	110.76 (18)	H29A—C29—H29B	109.5
C36—C34—C35	111.3 (2)	C27—C29—H29C	109.5
N2—C34—H34	108.3	H29A—C29—H29C	109.5
C36—C34—H34	108.3	H29B—C29—H29C	109.5
C35—C34—H34	108.3	C27—C28—H28A	109.5
C21—C23—H23A	109.5	C27—C28—H28B	109.5
C21—C23—H23B	109.5	H28A—C28—H28B	109.5
H23A—C23—H23B	109.5	C27—C28—H28C	109.5
C21—C23—H23C	109.5	H28A—C28—H28C	109.5
H23A—C23—H23C	109.5	H28B—C28—H28C	109.5
H23B—C23—H23C	109.5	C34—C36—H36A	109.5
C5—C3—C6	107.14 (17)	C34—C36—H36B	109.5
C5—C3—C4	109.82 (17)	H36A—C36—H36B	109.5
C6—C3—C4	107.07 (18)	C34—C36—H36C	109.5
C5—C3—C2	111.19 (17)	H36A—C36—H36C	109.5
C6—C3—C2	111.92 (16)	H36B—C36—H36C	109.5
C4—C3—C2	109.58 (16)	C9—C10—H10A	109.5
C3—C6—H6A	109.5	C9—C10—H10B	109.5
C3—C6—H6B	109.5	H10A—C10—H10B	109.5
H6A—C6—H6B	109.5	C9—C10—H10C	109.5
C3—C6—H6C	109.5	H10A—C10—H10C	109.5
H6A—C6—H6C	109.5	H10B—C10—H10C	109.5
H6B—C6—H6C	109.5	C9—C12—H12A	109.5
C25—C20—C19	118.17 (17)	C9—C12—H12B	109.5
C25—C20—C21	121.55 (16)	H12A—C12—H12B	109.5
C19—C20—C21	120.28 (16)	C9—C12—H12C	109.5
C26—C31—C32	122.13 (18)	H12A—C12—H12C	109.5
C26—C31—H31	118.9	H12B—C12—H12C	109.5
C32—C31—H31	118.9	C9—C11—H11A	109.5
C21—C22—H22A	109.5	C9—C11—H11B	109.5
C21—C22—H22B	109.5	H11A—C11—H11B	109.5
H22A—C22—H22B	109.5	C9—C11—H11C	109.5
C21—C22—H22C	109.5	H11A—C11—H11C	109.5
H22A—C22—H22C	109.5	H11B—C11—H11C	109.5
			
O2—Zn1—O1—C1	117.19 (15)	Zn1—N2—C34—C35	72.3 (2)
N1—Zn1—O1—C1	−4.09 (16)	C7—C2—C3—C5	121.9 (2)
N2—Zn1—O1—C1	−134.71 (14)	C1—C2—C3—C5	−59.4 (2)
O1—Zn1—N2—C33	−122.85 (14)	C7—C2—C3—C6	2.1 (3)
O2—Zn1—N2—C33	−5.58 (15)	C1—C2—C3—C6	−179.21 (18)
N1—Zn1—N2—C33	120.64 (14)	C7—C2—C3—C4	−116.5 (2)
O1—Zn1—N2—C34	52.74 (16)	C1—C2—C3—C4	62.2 (2)
O2—Zn1—N2—C34	170.01 (15)	O2—C19—C20—C25	−177.27 (17)
N1—Zn1—N2—C34	−63.77 (16)	C32—C19—C20—C25	3.2 (3)
O1—Zn1—N1—C15	0.81 (15)	O2—C19—C20—C21	2.6 (3)
O2—Zn1—N1—C15	−117.16 (14)	C32—C19—C20—C21	−176.92 (16)
N2—Zn1—N1—C15	125.89 (14)	C22—C21—C20—C25	−1.1 (3)
O1—Zn1—N1—C16	173.28 (14)	C24—C21—C20—C25	−120.7 (2)
O2—Zn1—N1—C16	55.31 (16)	C23—C21—C20—C25	117.7 (2)
N2—Zn1—N1—C16	−61.64 (16)	C22—C21—C20—C19	178.94 (17)
O1—Zn1—O2—C19	128.66 (15)	C24—C21—C20—C19	59.4 (2)
N1—Zn1—O2—C19	−122.00 (15)	C23—C21—C20—C19	−62.2 (2)
N2—Zn1—O2—C19	8.93 (16)	C19—C20—C25—C26	−1.3 (3)
Zn1—O1—C1—C14	7.0 (3)	C21—C20—C25—C26	178.82 (18)
Zn1—O1—C1—C2	−172.01 (12)	C14—C13—C8—C7	−0.2 (3)
Zn1—O2—C19—C32	−5.2 (3)	C14—C13—C8—C9	178.99 (18)
Zn1—O2—C19—C20	175.25 (12)	C2—C7—C8—C13	−0.9 (3)
O1—C1—C2—C7	−179.46 (17)	C2—C7—C8—C9	179.93 (18)
C14—C1—C2—C7	1.5 (3)	C13—C8—C9—C11	−123.5 (3)
O1—C1—C2—C3	1.8 (3)	C7—C8—C9—C11	55.6 (3)
C14—C1—C2—C3	−177.24 (16)	C13—C8—C9—C12	−2.5 (3)
C1—C2—C7—C8	0.2 (3)	C7—C8—C9—C12	176.6 (2)
C3—C2—C7—C8	178.92 (18)	C13—C8—C9—C10	116.3 (3)
O1—C1—C14—C13	178.48 (17)	C7—C8—C9—C10	−64.6 (3)
C2—C1—C14—C13	−2.5 (3)	C32—C31—C26—C25	2.3 (3)
O1—C1—C14—C15	−6.2 (3)	C32—C31—C26—C27	−176.98 (18)
C2—C1—C14—C15	172.80 (17)	C20—C25—C26—C31	−1.5 (3)
C1—C14—C13—C8	1.9 (3)	C20—C25—C26—C27	177.80 (18)
C15—C14—C13—C8	−173.86 (17)	C31—C26—C27—C30	−3.3 (3)
C16—N1—C15—C14	−173.53 (18)	C25—C26—C27—C30	177.48 (19)
Zn1—N1—C15—C14	−0.6 (3)	C31—C26—C27—C28	117.3 (2)
C13—C14—C15—N1	178.47 (19)	C25—C26—C27—C28	−62.0 (2)
C1—C14—C15—N1	2.9 (3)	C31—C26—C27—C29	−123.7 (2)
C15—N1—C16—C17	−130.34 (19)	C25—C26—C27—C29	57.1 (2)
Zn1—N1—C16—C17	57.0 (2)	O2—C19—C32—C31	178.00 (17)
C15—N1—C16—C18	107.4 (2)	C20—C19—C32—C31	−2.5 (3)
Zn1—N1—C16—C18	−65.2 (2)	O2—C19—C32—C33	−4.2 (3)
C34—N2—C33—C32	−176.93 (19)	C20—C19—C32—C33	175.34 (17)
Zn1—N2—C33—C32	−1.0 (3)	C26—C31—C32—C19	−0.3 (3)
C33—N2—C34—C36	124.6 (2)	C26—C31—C32—C33	−178.31 (18)
Zn1—N2—C34—C36	−51.1 (2)	N2—C33—C32—C19	7.7 (3)
C33—N2—C34—C35	−112.0 (2)	N2—C33—C32—C31	−174.42 (19)

### Hydrogen-bond geometry (Å, º)

**Table:**

*D*—H···*A*	*D*—H	H···*A*	*D*···*A*	*D*—H···*A*
C4—H4*A*···O1	0.98	2.37	3.018 (3)	123
C5—H5*C*···O1	0.98	2.32	2.967 (3)	123
C23—H23*C*···O2	0.98	2.35	2.994 (3)	122
C24—H24*A*···O2	0.98	2.33	2.986 (3)	124

## References

[bb1] Bruker (2007). *APEX2*, *SAINT* and *SADABS*. Bruker AXS Inc., Madison, Wisconsin, USA.

[bb2] Chamberlain, B. M., Cheng, M., Moore, D. R., Ovitt, T. M., Lobkovsky, E. B. & Coates, G. W. (2001). *J. Am. Chem. Soc.* **123**, 3229–3238. 10.1021/ja003851f 11457057

[bb3] Chen, H.-Y., Tang, H.-Y. & Lin, C.-C. (2006). *Macromolecules*, **39**, 3745–3752.

[bb4] Chisholm, M. H., Gallucci, J. C., Zhen, H. & Huffman, J. C. (2001). *Inorg. Chem.* **40**, 5051–5054. 10.1021/ic010560e 11531458

[bb5] Sheldrick, G. M. (2008). *Acta Cryst.* A**64**, 112–122. 10.1107/S0108767307043930 18156677

[bb6] Westrip, S. P. (2010). *J. Appl. Cryst.* **43**, 920–925.

[bb7] Wheaton, C. A. & Hayes, P. G. (2011). *Comments Inorg. Chem.* **32**, 127–162.

[bb8] Williams, C. K., Breyfogle, L. E., Choi, S. K., Nam, W., Young, V. J. Jr, Hillmyer, M. A. & Tolman, W. B. (2003). *J. Am. Chem. Soc.* **125**, 11350–11359. 10.1021/ja0359512 16220958

[bb9] Wu, J.-C., Yu, T.-L., Chen, C.-T. & Lin, C.-C. (2006). *Coord. Chem. Rev.* **250**, 602–626.

