# Supplementary figures and images for: Crystal structure of bis­{2,4-di-tert-butyl-6-[(iso­propyl­imino)­meth­yl]phenolato-κ2 N,O}zinc di­chloro­methane mono­solvate
Source: Acta Crystallogr Sect E Struct Rep Online. 2014 Nov 5;70(Pt 12):m390–1. doi: 10.1107/S1600536814022636 (PMC4257443; doi:10.1107/S1600536814022636)

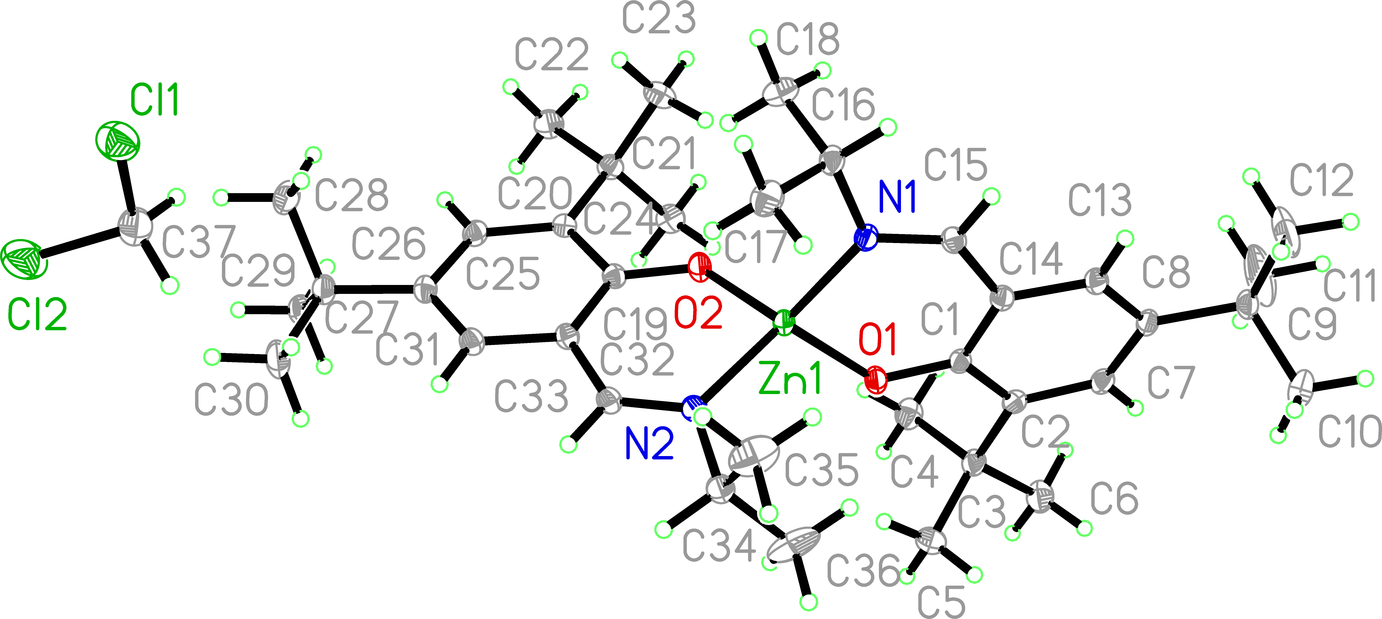

Supplement: Supplementary file 3 [file e-70-0m390-fig1.tif]

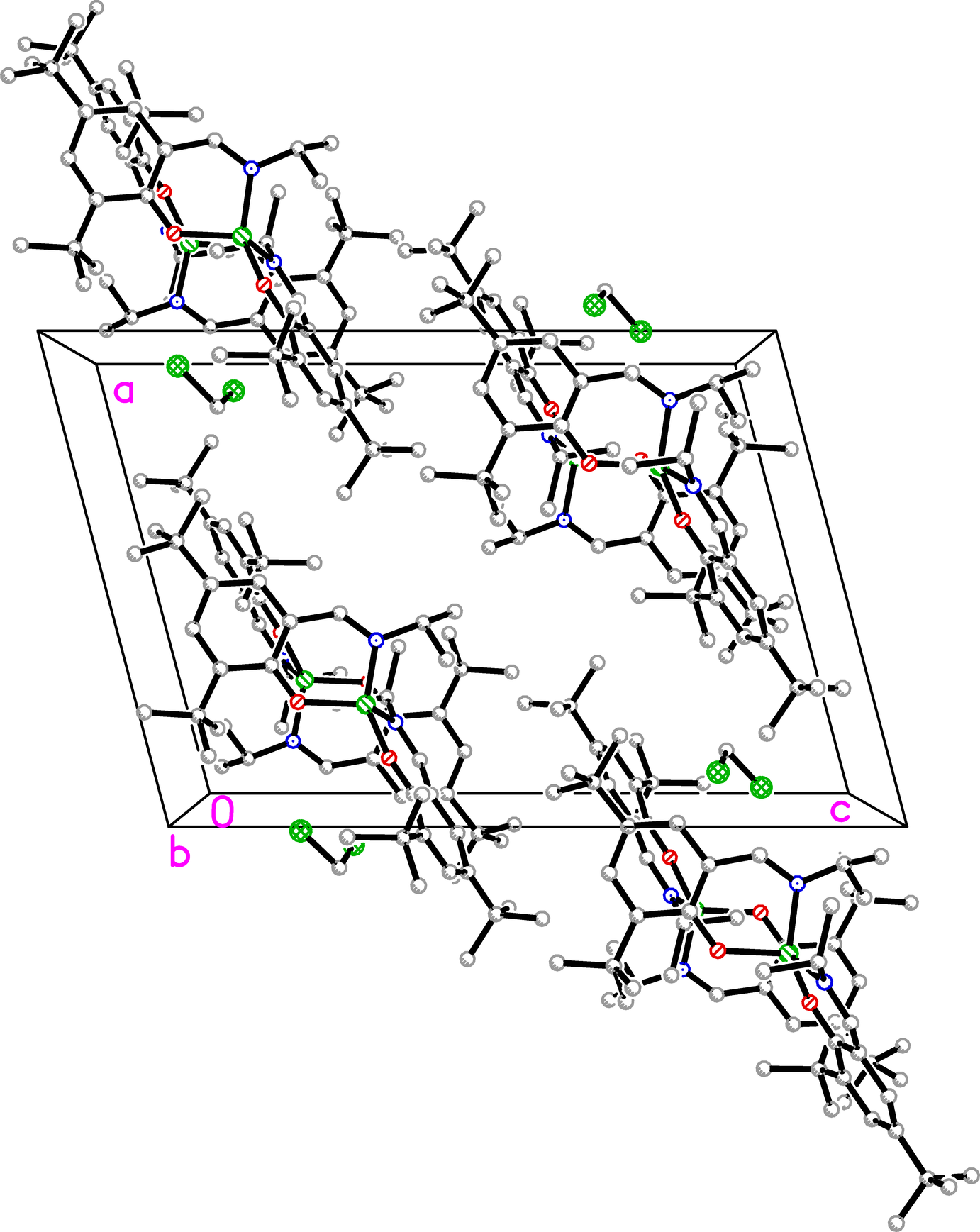

Supplement: Supplementary file 4 [file e-70-0m390-fig2.tif]
